# A separable temporal convolutional networks based deep learning technique for discovering antiviral medicines

**DOI:** 10.1038/s41598-023-40922-y

**Published:** 2023-08-22

**Authors:** Vishakha Singh, Sanjay Kumar Singh

**Affiliations:** Department of Computer Science and Engineering, Indian Institute of Technology (BHU) Varanasi, Varanasi, Uttar Pradesh 221005 India

**Keywords:** Computational biology and bioinformatics, Computational models, Protein analysis, Virtual drug screening

## Abstract

An alarming number of fatalities caused by the COVID-19 pandemic has forced the scientific community to accelerate the process of therapeutic drug discovery. In this regard, the collaboration between biomedical scientists and experts in artificial intelligence (AI) has led to a number of *in silico* tools being developed for the initial screening of therapeutic molecules. All living organisms produce antiviral peptides (AVPs) as a part of their first line of defense against invading viruses. The Deep-AVPiden model proposed in this paper and its corresponding web app, deployed at https://deep-avpiden.anvil.app, is an effort toward discovering novel AVPs in proteomes of living organisms. Apart from Deep-AVPiden, a computationally efficient model called Deep-AVPiden (DS) has also been developed using the same underlying network but with point-wise separable convolutions. The Deep-AVPiden and Deep-AVPiden (DS) models show an accuracy of 90% and 88%, respectively, and both have a precision of 90%. Also, the proposed models were statistically compared using the Student’s t-test. On comparing the proposed models with the state-of-the-art classifiers, it was found that they are much better than them. To test the proposed model, we identified some AVPs in the natural defense proteins of plants, mammals, and fishes and found them to have appreciable sequence similarity with some experimentally validated antimicrobial peptides. These AVPs can be chemically synthesized and tested for their antiviral activity.

## Introduction

The discovery of novel antimicrobial drugs that kill or inhibit life-threatening pathogens is attracting much attention due to the incapacity and inefficiency of conventional antibiotics. However, it is pertinent that the new class of therapeutics must have high efficacy, broad-spectrum activity, and few or no side effects on human health. In this direction, medications can be developed using antimicrobial peptides (AMPs), which form an integral part of living organisms’ natural first line of defense. Nowadays, analyzing and modeling AMPs using machine/deep learning has caught momentum^1–7^. Deep learning-based sequence modeling techniques such as recurrent neural networks (RNNs), long-short term memory (LSTM) networks, temporal convolutional networks (TCNs)^8,9^, etc., can be effectively used to develop robust models to classify and discover novel therapeutic peptides like AMPs, anti-cancer peptides^10,11^, etc., in proteomes of various life-forms. Note that sequence modeling is a technique that inputs and outputs sequential data, which can be in the form of text, audio, video, etc. For this purpose, RNN was developed as a deep learning architecture for capturing dependencies between the units of a given sequence to make predictions. However, it fails to capture long-range dependencies between these units due to the vanishing gradient problem. LSTMs were proposed as an improvement over RNNs in that they overcome this problem by using a gating mechanism (input, output, and forget gates) to remember the correlation among the units over a long range. However, LSTMs require more memory than RNNs to store partial results. Additionally, RNN and LSTM-based models work sequentially, so the units of a given sequence (also known as timesteps) cannot be processed in parallel. However, such shortcomings are not present in TCNs. The computations performed by this deep learning architecture can be easily distributed and parallelized on multi-core processing systems, and it also does not consume much memory.

Several models have been built to classify antiviral peptides (AVPs) using these deep learning algorithms. Note that AVPs are a sub-class of AMPs that target the host against invading viruses by targeting them or the host cells to inhibit viral replication. Some AVPs are virucidal because they either inhibit the viral protein outside the host cell or compete for the link-site on the host’s cell membrane^12^. In contrast, some others interfere with different stages of the viral life cycle, such as viral gene expression, replication, etc. Interestingly, numerous AVPs are present in the proteomes of mammals, plants, fishes, and other living organisms. A family of AVPs called cyclotides found in plants prevents a wide array of human viruses such as human immunodeficiency virus (HIV)^13^, H1N1^14^, and dengue^15^ from binding to the host’s cell membrane. The Cecropin-A derived from a moth acts against HIV by suppressing its genetic expression. Similarly, a family of antimicrobial peptides (AMPs) known as dermaseptins found in the frogs of the Phyllomedusa genus have shown virucidal potential against HIV-1^16^. Speaking of AVPs derived from marine organisms, a class of peptides known as clavanins inhibits the virulence of herpes simplex virus (HSV), rotavirus, and adenovirus^17^.

Some of the deep learning-based tools built to classify AVPs are as follows. The Deep-AVPpred model uses convolutional neural networks (CNNs) for the prediction and discovery of AVPs^18^, while DeepAVP^19^ uses both bidirectional-LSTM and CNN for the same. In^20^, the authors performed multi-label classification for predicting several functional activities exhibited by a peptide (antiviral, anti-HIV, antibacterial, antifungal, etc.) using bi-LSTM, CNN, and support vector machine (SVM). Lastly, the authors of^21^ trained various machine/ deep learning architectures like the Transformers, CNNs, bi-LSTM, Random Forests (RFs), and Support Vector Machine (SVM) on a set of AVPs and found RF with Word2Vec representations to be the highest-performing model (iACVP) to predict anti-coronavirus peptides. Deep learning architectures like CNN fail to capture long-range dependencies between the units of an AVP, i.e., the amino acids (AAs). The bi-LSTM-based models evade this drawback but not in the case of very long sequences. Also, training and tuning a bi-LSTM model takes considerable time (due to its sequential execution and non-parallelizable architecture) and consumes a lot of memory, too^22^. To sum up, a significant issue deep neural networks face is the computationally expensive mode of training and operation. In other words, deep learning models consume a lot of computational resources while getting trained. They are large in size, making their training and deployment very difficult in resource-constrained environments.

Apart from the deep learning algorithms, researchers have been using some quantifiable properties of peptides (known as their physicochemical, compositional, and structural properties) with machine learning algorithms like SVM, random forests (RFs), etc., to build AVP classifiers. The authors of^23^ used several hand-engineered features derived from peptide sequences, i.e., motifs, amino acid composition, and some physicochemical properties, to classify AVPs. The AntiVPP 1.0 model uses the RF algorithm that uses compositional and physicochemical features to predict antiviral peptides. Pang et al.^24^ proposed the AVPiden model based on RFs to perform a two-stage classification. In the first stage, it categorizes peptides as AVPs and non-AVPs, and in the second stage, it predicts the potential of AVPs against eight kinds of viruses and six virus families. In^25^, the authors employed four machine learning algorithms, namely, SVMs, RFs, Instance-based classifier, and K-star, to perform AVP classification using physicochemical properties with amino-acid composition, the binary profile of residues, etc. The ENNAVIA model^26^ uses physicochemical and compositional features on a deep neural network architecture for classifying AVPs and non-AVPs. In yet another study^27^, authors used six machine learning algorithms for this purpose. The PreTP-Stack model is built using ten features and four machine learning algorithms^28^. Lastly, in the FIRM-AVP model^29^, Chowdhury et al. used three machine learning techniques for building an AVP classifier and found that the SVM-based model performs the best. The biggest drawback of using machine learning-based models is the added burden of crafting, collecting, and refining hand-engineered features that serve as the input. Also, the machine learning models lag behind their deep learning counterparts in terms of performance when the dataset is large. Another shortcoming of these studies is that most of them do not have dedicated web servers to help wet lab researchers discover and classify AVPs^30^.

To overcome most of the aforementioned shortcomings, we propose a model based on TCNs^8,9,22^ named Deep-AVPiden for the classification and discovery of AVPs. The TCNs are abundantly employed for sequence modeling since they are faster than bi-LSTM networks and can also easily capture long-range dependencies, unlike CNNs. The proposed model is trained to identify AVPs in proteins of various organisms like mammals, plants, amphibians, fishes, arthropods, etc. The model’s performance has been compared with the existing state-of-the-art classifiers like AVPIden, ENNAVIA, iAMP-CA2L, Meta-iAVP, PreTP-Stack, iACVP, and DeepAVP, and the results indicate that it performs better than them. Moreover, we also proposed an alternate model using depth-wise separable convolutions that drastically reduces the number of training parameters as compared to standard convolutions. The model that is trained using this technique is named Deep-AVPiden (DS). A web app has been built using both models and deployed at https://deep-avpiden.anvil.app/. Apart from classifying AVPs, this app can also discover AVPs in proteins. To illustrate the working of this app, we found some AVPs in several antiviral proteins found in mammals, plants, and fishes. This paper’s major contributions are enumerated as follows.

1. A novel deep learning model based on TCNs called Deep-AVPiden has been proposed to distinguish between AVPs and non-AVPs.
2. An alternate model called Deep-AVPiden (DS), which is a less compute and memory-intensive version of Deep-AVPiden, has also been proposed using point-wise separable TCNs. This model can be easily deployed on resource-constrained devices for discovering AVPs.
3. A web app based on these models has been built and deployed at https://deep-avpiden.anvil.app/ with the ability to discover AVPs in protein sequences to aid wet-lab researchers.
4. Using the app, 15 AVPs were identified in proteins of plants, mammals, and fishes and proposed for chemical synthesis and experimental validation.
5. The proposed models have been compared with the state-of-the-art classifiers and shown to perform better than them.
6. The proposed models were statistically analyzed and compared using the Student’s t-test.

**Figure 1:**
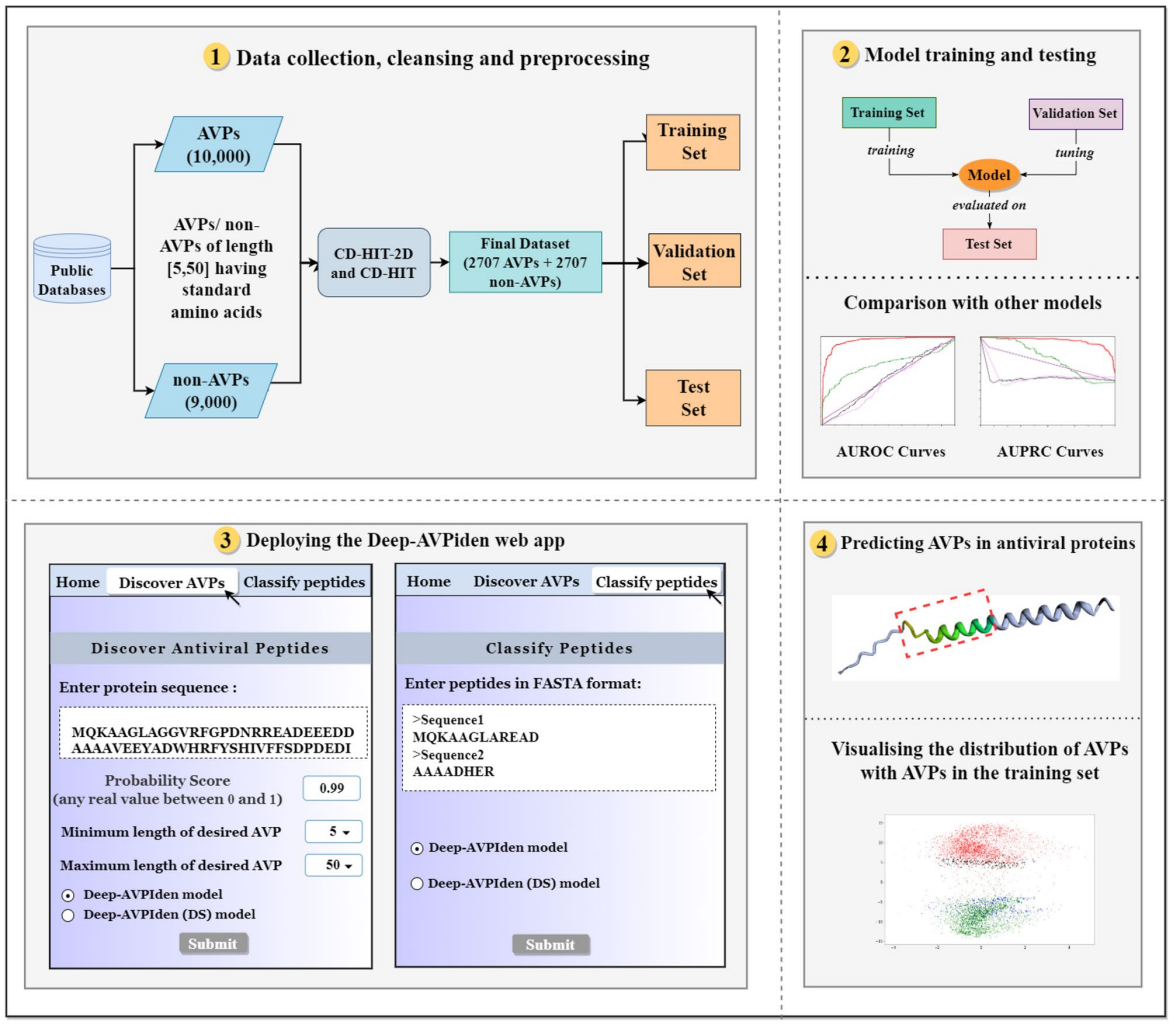
The layout of the proposed work.

The rest of the paper is depicted through Fig. 1 and organized as follows. Section “Materials and methods” briefly describes the dataset and methods used to build the proposed model, Deep-AVPiden. Section “Proposed model” contains a detailed description of our model. Section “Results and discussions” comprises comparing the proposed models with each other and the existing state-of-the-art classifiers. Here, we have also presented the AVPs predicted in antiviral proteins of plants, mammals, and fishes. Lastly, in section “Conclusion”, the concluding remarks and prospects of future works have been elucidated.

## Materials and methods

In this section, we describe the dataset and the sequence modeling technique that was used to build the Deep-AVPiden model.

### Dataset

The proposed models use peptides as data points which are basically alphabetical strings where each letter represents a standard amino acid. The AVPs were collected from various sources such as AVPdb^31^, HIPdb^32^, the starPep database^33–35^, DRAMP^36^, and the SATPdb^37^. The non-AVPs were taken from the Swiss-Prot database^38^ and AVPdb. After collecting 10,500 AVPs and 9000 non-AVPs, data cleansing was performed. Peptides that were composed of non-standard amino acids (*B*, *J*, *O*, *U*, *X*, and *Z*) and containing less than five or more than fifty amino acids were removed. Then we used CD-HIT^39–41^ program separately on the AVPs and non-AVPs with a threshold of 0.9 for filtering out similar sequences from the AVPs and non-AVPs, respectively. To eliminate any bias in performance due to the imbalance in the number of instances belonging to each class, we randomly removed 699 non-AVPs. The final dataset consisted of 5414 peptides (comprising 2707 AVPs and 2707 non-AVPs) which were then sub-divided into training (70% of the data points), test (15% of the data points), and validation (15% of the data points) sets.

### Data pre-processing

The data points, represented by alphabetical strings, were tokenized and converted into numerical strings using a one-to-one character-to-integer mapping. This was done to convert the input into a computer-understandable format. Since the dataset comprised numerical strings of varying lengths, to bring uniformity, the strings with lengths in the interval [5,49] were padded with extra zeroes until their lengths became equal to 50. This resulting set of numerical strings was trifurcated into training, validation, and test sets. Then, the training set was used to generate a word embedding matrix (thoroughly described in section “Proposed model”).

### Word embeddings

Word embedding techniques convert each word (the numbers representing amino acid residues) into a fixed-length vector. One-hot encoding (OHE), and word2vec are the most common methods used for this purpose. There are two popular word2vec algorithms: the skip-gram and continuous bag of words (CBoW), which convert each word (represented by a one-hot vector) into a fixed-length feature vector using its context (which refers to the words surrounding a given word in the data points of the training set). Thus, semantically similar words are given similar feature vector representations.

### Temporal convolutional networks

Temporal Convolutional Networks (TCNs) consist of one or more blocks of one-dimensional convolutional (1D-CONV) layers. In these layers, the filter taps may be applied on the input units or time steps in a non-consecutive manner. In other words, the dilated convolutions are used, in which case it is not necessary that in a given 1D-CONV layer, the filter taps must be applied on consecutive time steps. This is controlled by the size of the dilation factor (*d*), which increases the receptive field (which essentially means that the layers can capture dependencies between time steps over a long range).

Temporal convolutional networks are of two types: acausal and causal. In causal TCNs, a CONV layer uses only the past time steps (1 to *t*-1) to calculate the output at a time step *t*, whereas in acausal TCNs, the past and future units are used for this purpose. In this work, causal TCNs have been used to build the models. The convolution operation (*C*(*t*)) at position *t* in a dilated causal 1D-CONV layer with a dilation factor of *d* is given by Eq. (1)^22^.

1 $$\begin{aligned} C(t) = (x *_d f)(t)= \sum _{i=1}^{k}{f(i).x_{t-d.(i-1)}} \end{aligned}$$

Here, *x* is the input to the layer, $*_d$ is the convolution operation, and *f* is a 1D filter of size *k*. We may use skip-connections in a TCN block, which are known to prevent the problem of vanishing/exploding gradients and can even be used to prevent the degradation problem and overfitting^42^. Each residual block comprises two 1D-CONV layers, and a skip connection is introduced by adding a block’s input with its output. This converts a regular TCN block into a residual TCN block whose output (*y*) is as per the given equation.

2 $$\begin{aligned} y = activation(x + F(x)) \end{aligned}$$

Here, *F*(*x*) is the output of the last layer of the TCN block, and *activation* is a non-linear function (e.g., ReLU). Skip connections allow the residual block to learn an identity function of the input, which may help stabilize the learning process in deep neural networks.

### Depth-wise separable convolutions

The concept of depth-wise separable convolutions (DwSCs) came into existence due to the rising interest of the research community in building small and efficient models. Before the conception of this idea, either the pre-trained models were compressed or the underlying networks were made shallow. Thus, as an alternative, DwSCs were introduced in^43^ and later successfully used in^44,45^ to train deep ConvNets. It factorizes a standard convolution operation into two parts, i.e., depth-wise and point-wise convolutions, described as follows.

1. **Depth-wise Convolutions**: In this stage, a single filter is applied to every input channel separately. So, if in a standard convolution operation, we had to apply *N* filters of size 1 X $f_k$ X $n_c$ ($f_k$ is the specified filter size and $n_c$ is the number of channels) on a 2-D matrix of size 1 X *M* X $n_c$, only one filter of size 1 X $f_k$ X 1 would be applied on $n_c$ channels separately to get an output of size 1 X $M'$ X $n_c$.
2. **Point-wise Convolutions**: A 1 X 1 convolutional layer comprising *N* filters of size 1 X 1 X $n_c$ is applied on the output of the depth-wise convolutions. IT=t gives an output of size 1 X $M'$ X *N*.

This kind of factorization reduces the number of training parameters used in the network, which leads to a considerable reduction in the number of computations while the model training phase. The resultant model gets trained in less time, consumes less space, and can be efficiently trained and deployed on resource-constrained platforms, including mobile devices.

## Proposed model

**Figure 2:**
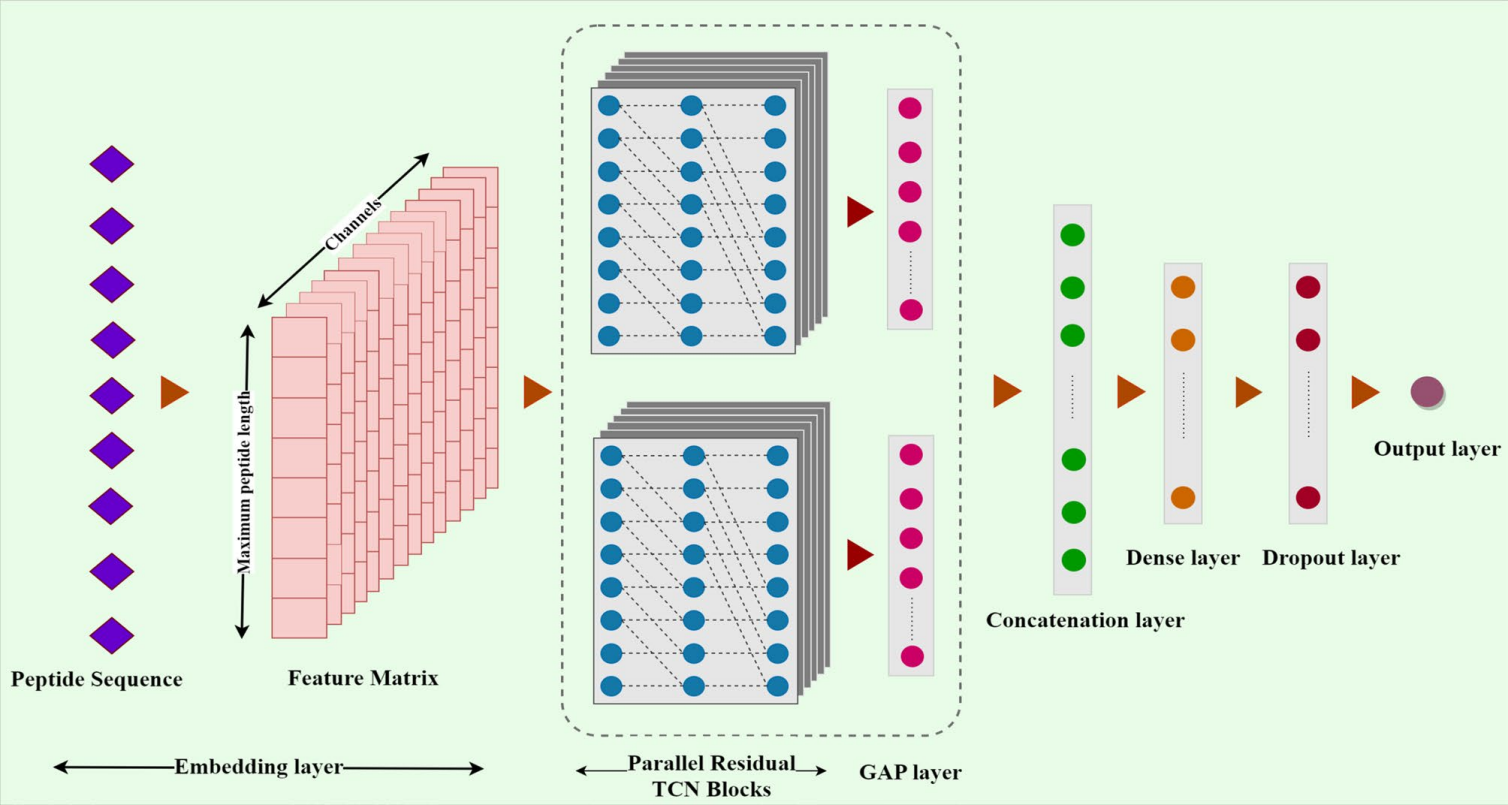
The deep-AVPiden architecture.

As shown in Fig. 2, the Deep-AVPiden model consists of many layers described as follows.

1. **Embedding layer**: In this work, the skip-gram algorithm has been used to construct a word embedding matrix for 20 standard amino acid residues using the data points contained in our training set. This layer converts the numerical string into a (50,512) feature matrix (where the first element indicates the size of each numerical string, and the other element is the size of the fixed-length feature vector representation of an amino acid).
2. **Spatial Dropout layer**: This layer performs regularization by dropping columns (frames) from the feature matrix instead of individual elements. This layer is preferred over the normal dropout layer when the correlation between the frames is high. The Deep-AVPiden model employs a 1D spatial dropout layer after the embedding layer( with a dropout rate of 0.5).
3. **TCN blocks**: This model uses dilated causal TCN architecture. It contains two parallel TCN blocks consisting of 1D-CONV layers with one skip connection. The difference between the two blocks lies in the size of the filters used in them. These layers employed rectified linear unit (ReLU) as the activation function. We used batch normalization layers in between layers to stabilize the learning process. As far as the dilation factor *d* is concerned, it increases in the consecutive layers by a factor of 2 (we have used d= 1, 2, and 4).
4. **Global Average Pooling (GAP) layer**: A 1D-GAP layer is used after each TCN block. It computes the average of the feature map obtained from the TCN blocks.
5. **Concatenation layer**: It simply combines the output of the two GAP layers for further processing.
6. **Dense layer with dropout**: A dense layer has been incorporated after the concatenation layer. It contains 64 units and uses ReLU as its activation function. After this layer, a dropout layer is incorporated to prevent overfitting.
7. **Output layer**: This layer consists of a neuron using the sigmoid function for activation. The output of this neuron is a real number lying in the interval [0,1]. A peptide is predicted as an AVP if the output is greater than or equal to 0.5.

Apart from training the model using standard convolutions, depth-wise separable convolutions were used for building a more efficient model (in terms of computation and storage space). In other words, two models have been proposed in which one model comprises residual TCN networks that use standard convolutions. In contrast, the other was built by replacing the 1D-CONV layers in the TCN blocks with depth-wise and point-wise convolutional layers. These models have been compared and discussed in section “Results and discussions”.

## Results and discussions

This section presents the details about the setup used to train the models, followed by their comparison with the state-of-the-art models based on the specified performance metrics. Also, a pilot study of the free web app has been done and elaborated using some representative protein sequences found in various organisms.

### Experimental setup

The proposed models were trained on a compute node having 2.4 GHz Intel-Xeon Skylake 6148 CPU processors with RAM of 192 GB and NVIDIA V100 graphical processing units with 16 GB RAM. We used Python for coding and certain libraries such as Keras with Tensorflow^46^ as the backend and Keras-TCN library^47^. These models were compared using a test set with various state-of-the-art classifiers like DeepAVP^19^, AVPIden^24^, iAMP-CA2L^20^, ENNAVIA^26^, Meta-iAVP^27^, PreTP-Stack^28^, and iACVP^21^. Note that we have compared with only those models that have removed identical and homologous sequences from their dataset, which is important to prevent any bias in a model’s performance. Furthermore, ENNAVIA and AVPIden, only classify sequences with lengths lying in the interval [7,40] and [8,50], respectively. Also, iACVP classifies sequences having more than 5 AA residues. So, when these models were executed, the test set was curated as per their specifications. Also, after obtaining the results of iAMP-CA2L, it was observed that this model sometimes does not label the functional type of an AMP (i.e., whether the classified AMP is antibacterial or antiviral, etc.). So, to prevent any ambiguity, we removed such instances from the test set while giving the results for iAMP-CA2L.

### Performance metrics

The models were compared on certain performance metrics like accuracy, precision, and the area under the receiver operating characteristic curve (AUC-ROC). All of these metrics can be expressed in terms of True Positives (TPs, or the number of AVPs that were correctly identified), False Positives (FPs, or the number of non-AVPs that were incorrectly identified as AVPs), True Negatives (TNs, or the number of non-AVPs that were correctly identified), False Negatives (FNs, or the number of AVPs that were incorrectly identified as non-AVPs). It is evident that the Deep-AVPiden model outperforms other models by a significant margin.

3 $$\begin{aligned} Accuracy= {} \frac{TP+TN}{TP+TN+FP+FN} \end{aligned}$$

4 $$\begin{aligned} Precision= {} \frac{TP}{TP+FP} \end{aligned}$$

5 $$\begin{aligned} Recall \text{ (or} \,\textit{True Positive Rate}\, (\textit{TPR}))= {} \frac{TN}{TN+FP} \end{aligned}$$

6 $$\begin{aligned} {\textit{False Positive Rate} (\textit{FPR})}= {} 1-\frac{TN}{FP+TN} \end{aligned}$$

7 $$\begin{aligned} AUC-ROC= {} \int TPR. \text{ d}(FPR) \end{aligned}$$

### Performance evaluation and comparison

**Figure 3:**
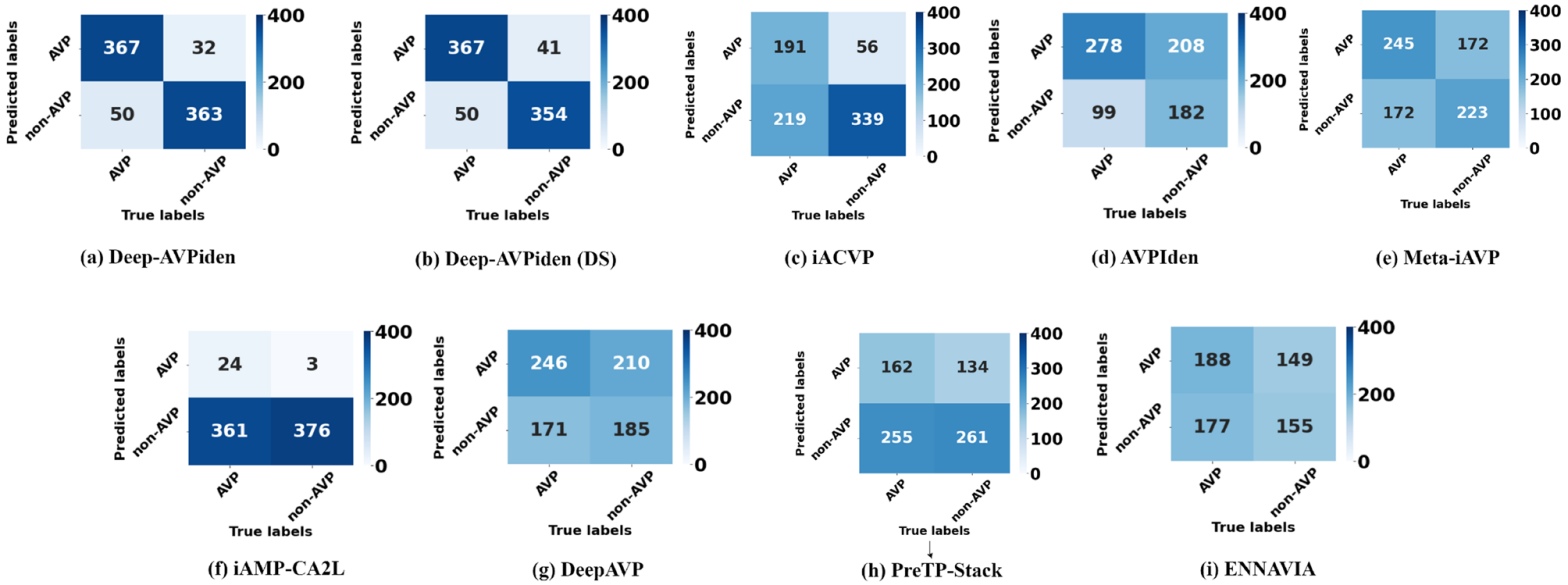
Confusion matrices obtained for various models including Deep-AVPiden on the test set.

While building the model, both causal and acausal TCNs were considered. However, there was not much difference in their performance, as mentioned in Table 1, and the mean accuracy, recall, and AUROC of the model built using the causal convolutions was higher than its acausal counterpart. Hence, causal TCNs were used to build the Deep-AVPiden model. The results of performance given by various state-of-the-art models, including Deep-AVPiden and Deep-AVPiden (DS), have been presented in Table 2. It is evident that both models outperform others by a significant margin with respect to all the performance metrics. The confusion matrices for different models have been shown in Fig. 3. Here, it is observable that the proposed models give more TPs and TNs and fewer FPs and FNs than others.

**Table 1:** Comparison between acausal and causal TCNs considered while building the model.

Model	Accuracy (%)	Precision (%)	Recall (%)	AUROC (%)
Deep-AVPiden (causal)	89.88± 0.01	90.29 ± 1.74	90.09 ± 1.72	95.99 ± 0.01
Deep-AVPiden (acausal)	89.77± 0.38	90.55 ± 1.32	88.73 ± 1.89	95.89 ± 0.31

**Table 2:** Comparison of deep-AVPiden with existing models on test set.

Model	Accuracy (%)	Precision (%)	Recall (%)	AUROC (%)
Deep-AVPiden	89.88± 0.00	90.29 ± 1.74	90.09 ± 1.72	95.99 ± 0.01
Deep-AVPiden (DS)	88.47±0.13	88.49 ± 0.40	88.98 ± 0.38	94.90 ± 0.05
iACVP	65.83	77.33	46.59	75.49
AVPIden	59.98	57.20	73.74	68.81
Meta-iAVP	57.63	58.75	58.75	58.29
DeepAVP	53.08	53.94	58.99	52.77
iAMP-CA2L	52.36	88.89	6.23	52.72
PreTP-Stack	52.09	54.73	38.85	52.46
ENNAVIA	51.27	55.79	51.51	48.99

Apart from this, an insight into the pros and cons of both models is also required. As is clear from Fig. 3, the Deep-AVPiden performs better than the Deep-AVPiden (DS) model. To check the statistical significance of the difference in the performance of the proposed models, the Student’s t-test has been employed. The null ($H_0$) and alternate ($H_1$) hypotheses’ are given in Eqs. (8–9). Table 3 presents the results of this statistical test. Note that, in the t-test, if the p-value is lesser than the chosen alpha level (here, 0.05), then it can be claimed that there is a difference between the means of the classifiers under comparison.

8 $$\begin{aligned} {H_0 ({accuracy})}: \mu _{accuracy} (Deep-AVPiden)&= \mu _{accuracy} (Deep-AVPiden (DS))\\ {H_0 ({precision})}: \mu _{precision} (Deep-AVPiden)&= \mu _{precision} (Deep-AVPiden (DS))\\ {H_0 ({recall})}: \mu _{recall} (Deep-AVPiden)&= \mu _{recall} (Deep-AVPiden (DS))\\ {H_0 ({AUC})}: \mu _{AUC} (Deep-AVPiden)&= \mu _{AUC} (Deep-AVPiden (DS))\\ \end{aligned}$$

9 $$\begin{aligned} {H_1 ({accuracy})}: \mu _{accuracy} (Deep-AVPiden)&\ne \mu _{accuracy} (Deep-AVPiden (DS))\\ {H_1 ({precision})}: \mu _{precision} (Deep-AVPiden)&\ne \mu _{precision} (Deep-AVPiden (DS))\\ {H_1 ({recall})}: \mu _{recall} (Deep-AVPiden)&\ne \mu _{recall} (Deep-AVPiden (DS))\\ {H_1 ({AUC})}: \mu _{AUC} (Deep-AVPiden)&\ne \mu _{AUC} (Deep-AVPiden (DS))\\ \end{aligned}$$

Since the alpha level is greater than the p-value in the case of all the metrics, it can be said that $H_0 (accuracy), H_0 (precision), H_0 (recall), H_0 (AUC)$ are not true. In other words, the difference in means of all the performance metrics used to evaluate both models is statistically significant. There are other desirable attributes that need to be mentioned here. As mentioned in Table 4, the size and number of trainable parameters of Deep-AVPiden are approximately 2.5 times more than that of Deep-AVPiden (DS). Thus, although the latter lags a little behind the former in terms of performance, it is easily trainable and deployable on computationally-constrained devices. In other words, it consumes less computational resources and storage space. In conclusion, both the models have their own merits and hence can be used as per convenience and constraints of the environment in which they need to be invoked. E.g., if the server on which we want to deploy the model is a mobile phone, it is better to use Deep-AVPiden (DS). In all other cases, the Deep-AVPiden model can be used.

**Table 3:** Comparing Deep-AVPiden and Deep-AVPiden (DS) using t-test.

Observation	Deep-AVPiden	Deep-AVPiden (DS)
(a) t-test on accuracy (%) of proposed models		
Mean	89.879	88.466
Variance	0.002	0.132
Observations	10	10
Hypothesized Mean Difference	0	–
degrees of freedom	9	–
t-statistic	12.208	–
P (T$\le$t) one-tail	3.324E-07	–
t-Critical one-tail	1.833	–
P(T$\le$t) two-tail	6.648E-07	–
t-Critical two-tail	2.262	–
(b) t-test on precision (%) of proposed models		
Mean	90.289	88.494
Variance	1.737	0.403
Observations	10	10
Hypothesized Mean Difference	0	–
Degrees of freedom	13	–
t-Statistic	3.879	–
P(T$\le$t) one-tail	0.001	–
t-Critical one-tail	1.771	–
P(T$\le$t) two-tail	0.002	–
t-Critical two-tail	2.160	–
(c) t-test on recall (%) of proposed models		
Mean	90.098	88.984
Variance	1.719	0.379
Observations	10	10
Hypothesized Mean Difference	0	–
Degrees of freedom	13	–
t-Statistic	2.431	–
P(T$\le$t) one-tail	0.015	–
t-Critical one-tail	1.771	–
P(T$\le$t) two-tail	0.030	–
t-Critical two-tail	2.160	–
(d) t-test on AUC (%) of proposed models		
Mean	95.994	94.901
Variance	0.007	0.054
Observations	10	10
Hypothesized Mean Difference	0	–
Degrees of freedom	11	–
t-Statistic	14.044	–
P(T$\le$t) one-tail	1.136E-08	–
t-Critical one-tail	1.796	–
P(T$\le$t) two-tail	0.274E-08	–
t-Critical two-tail	2.201	–

**Table 4:** Comparison between Deep-AVPiden and Deep-AVPiden (DS) model.

S. no.	Deep-AVPiden	Deep-AVPiden (DS)
No. of training parameters	1.36 million	0.56 million
Size	15.70 MB	6.68 MB

### Predicting AVPs using Deep-AVPiden app

A freely accessible web app based on the Deep-AVPiden (and Deep-AVPiden (DS)) has been deployed at https://deep-avpiden.anvil.app. Using it, some AVPs have been discovered in the antiviral proteins of various mammals, plants, and fishes. These antiviral proteins belong to different families, including ribosome-inactivating protein (RIP), RNA-binding protein (RBP), and Dicer-like protein (DCL), among others. The RIPs have enzymatic activities (*N*-glycosidase, RNase, and DNase) that can damage ribosomes and interfere with protein translation. The RBPs, as the name suggests, bind to their target RNA and inhibit the translation and replication of RNA viruses. The proteins belonging to the DCL family perform RNA-silencing by cleaving double-stranded RNA (dsRNA) region of single-stranded RNAs (ssRNAs)^48^. The plant antiviral proteins belonging to these families, such as pokeweed antiviral protein (PAP), Phytolacca insularis antiviral protein (PIP), DCL4, Arabidopsis Pumilio-5 (APUM5), trichosanthin, etc., display antiviral activity against plant viruses such as Potato virus Y (PVY), Potato virus X (PVX), Cucumber mosaic virus (CMV), Potato leafroll virus (PLRV), Turnip mosaic virus (TuMV), etc.

**Table 5:** The AVPs discovered in antiviral proteins found in the proteomes of mammals, fish, and plants, with probability score $\ge$ 90% and showing some sequence similarity with the AMPs existing in public databases.

S.no	Accession number	Protein name	Protein length	Discovered AVPs
1.	AAS77872.1	PAP	313	SDPFETNKCRYHI
2.	AAD32679.1	PIP	315	FAPASTWAASPNPI
3.	NP_197532.3	DCL4	1702	LSCILNNLELLRSWK
4.	AAB31048.1	Trichosanthin	289	FISNLRKALPNERKLYDIPLL
5.	NP_001319600.1	APUM5	913	EELVKQLAGQMVSLSLQMYGCR
6.	AAI12003.1	IFN-alpha-1	189	ICSLGCDLPQTHSLAHT
7.	ABD52364.1	IFN-alpha-2	187	FCTEPSSAAWNRTL
8.	AAI19352.1	IFN-alpha-3	186	FTSKDLSATWNATLLDSF
9.	EAW58615.1	IFN-alpha-4	187	VLNCKSICSLGCDLPQ
10.	AAM78026.1	IFN-alpha-5	189	CNSVCSLGCDLPQTHGLL
11.	ATI15613.1	TRIM-8	568	LCPFCCISHCT
12.	KAG1939425.1	Ubl	379	RRSWPEPVIHPEPV
13.	AAO37934.1	Mx	626	PENIGEQIKRLIRKFI
14.	NP_001187107.1	IFN	162	FLNILNTRQLTELT
15.	TSK18011.1	PRDX1	417	FVILEKMLMEICVIFSCV

**Table 6:** .

AVP discovered		Similar annotated AMP				Similar AA positions with annotated AMPs
Sequence	No. of AAs	Sequence	No. of AAs	Validation method	Nature	
SDPFETNKCRYHI	13	VNT...QTT	262	X-Ray Diffraction	Antiviral	SDPFETNKCRYHI
FAPASTWAASPNPI	14	MET...GWF	224	Predicted (based on signature)	Antimicrobial	-AP-ST-A-SP-P
LSCILNNLELLRSWK	15	NWY...GIA	69	Predicted	Antimicrobial	L-CIL-N——–
FISNLRKALPNERKLYDIPLL	21	DVS...NMA	247	X-Ray Diffraction	Antiviral	FISNLRKALPNERKLYDIPLL
EELVKQLAGQMVSLSLQMYGCR	22	DDG...GSC	42	Predicted (based on signature)	Antimicrobial	—-K-LAGQM———–
ICSLGCDLPQTHSLAHT	17	CDL...SKE	165	Solution NMR	Antiviral	—–CDLPQTHSL—
FCTEPSSAAWNRTL	14	MAF...NSP	195	Experimentally validated	Antiviral	F-TE-SSAAW-TL
FTSKDLSATWNATLLDSF	18	CDL...SKE	165	Solution NMR	Antiviral	F–KD-SA-W-TLLD–
VLNCKSICSLGCDLPQ	16	GSV...TKD	31	Experimentally validated	Antiviral	VLNC—C-LG—–
CNSVCSLGCDLPQTHGLL	18	CDL...SKE	165	Solution NMR	Antiviral	——–CDLPQTH-L-
LCPFCCISHCT	11	QSH...CKF	25	Predicted	Antimicrobial	LC-FCC—–
RRSWPEPVIHPEPV	14	RRL...KPL	36	Predicted	Antimicrobial	-R-WP-P—P-P-
PENIGEQIKRLIRKFI	16	ELN...VEP	42	Predicted	Antimicrobial	-EN-GE-IK——-
FLNILNTRQLTELT	14	ATC...KGT	67	Predicted (based on signature)	Antimicrobial	——TRQLT-L-
FVILEKMLMEICVIFSCV	18	MHS...QNY	97	Predicted (based on signature)	Antimicrobial	F—E–L-E-C—SC-

The similar annotated peptides found using this tool are mentioned here. Column 5 shows the method used to validate these peptides as antimicrobial and/or antiviral (as mentioned in column 6). Lastly, column 7 consists of the similar AA positions between the discovered peptides and the ones found by BLAST analysis. Here ‘−’ represents dissimilarity between the amino acids present in the given peptides at that position.

**Figure 4:**
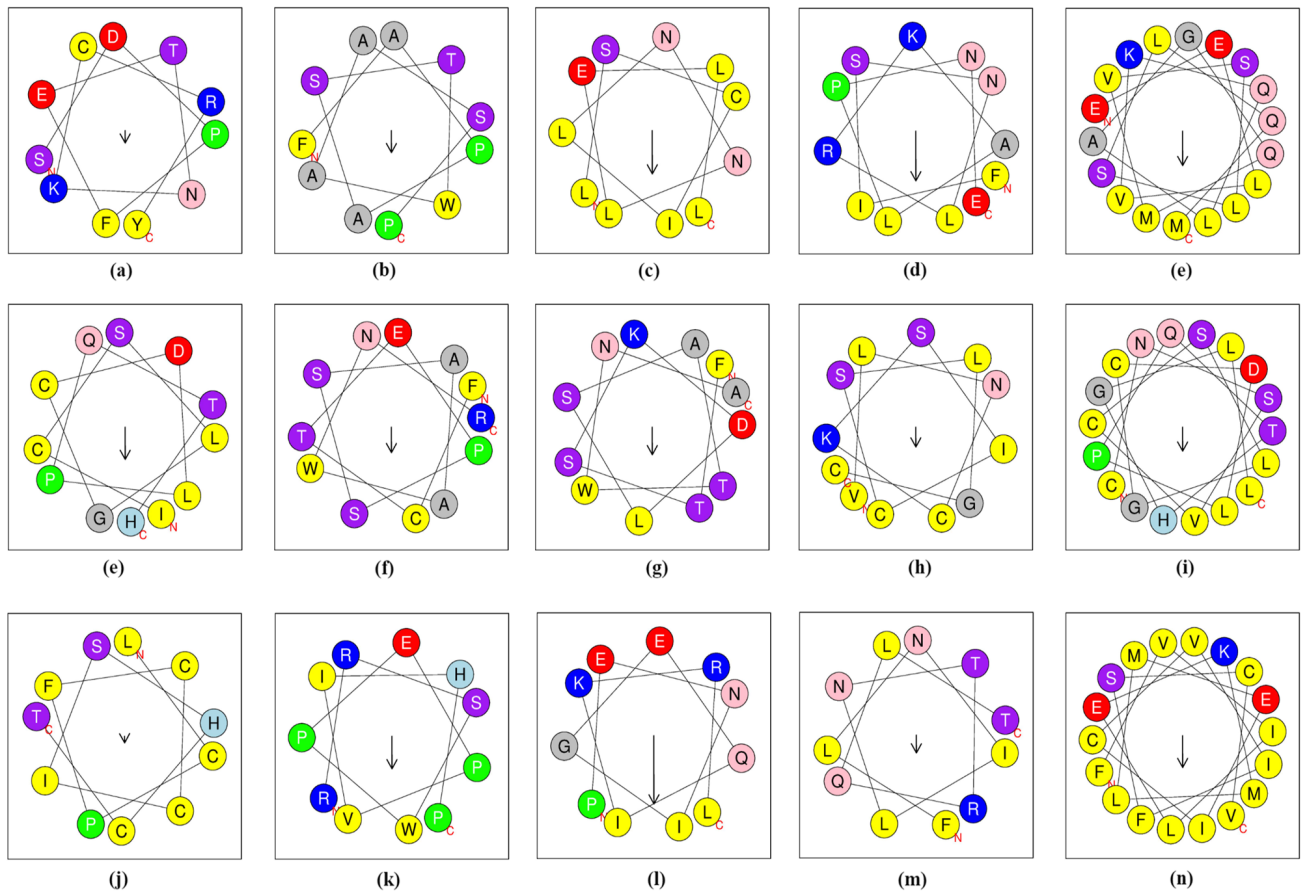
Alpha-helical representations of AVPs discovered in the plant, mammal, and fish proteins.

**Figure 5:**
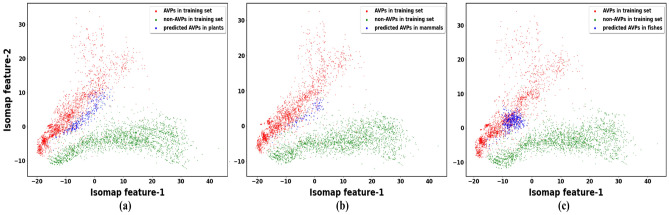
Scatter plots showing the distribution of AVPs predicted in the (**a**) plant, (**b**) mammal, and (**c**) fish antiviral proteins, along with the AVPs and non-AVPs in the training set.

The interferons (IFNs) are antiviral glycoproteins that can be classified (based on the structure of receptors on the cell surface) into three families: type I, II, and III IFNs. Proteins coded by the IFN-alpha genes have known antiviral activities, and they are actively used in the treatment of hepatitis C virus (HCV), hepatitis B virus (HBV), and human immunodeficiency virus-1 (HIV-1) infections^49–51^. Furthermore, Tripartite motif (TRIM), ubiquitin-like (Ubl), Peroxiredoxin-1 (PRDX1), and Mx proteins found in fishes use distinct mechanisms to inhibit entry, replication, and dissemination of HCV, HIV-1, etc.^52–54^. Some protein sequences mentioned in Table 5 were entered into the deployed web app with the following specifications.

1. Model: Deep-AVPiden (DS)
2. Probability Score: 0.90
3. Minimum length of desired AVPs : 10
4. Maximum length of desired AVPs : 30

After discovering AVPs in these proteins, the BLAST tool available at http://www.camp3.bicnirrh.res.in/ncbiBlast/^55^ was employed to confirm whether the discovered AVPs had any sequence similarity with existing experimentally validated AMPs present in public databases. Then, the AVPs with sufficient similarity with the annotated AMPs were selected and mentioned in Table 5. Apart from this, the method used for validating these AMPs and the similar AA positions with the discovered AVPs are highlighted in Table 6. The alpha-helical representations of these peptides have been shown in Fig. 4 using an online tool available at https://heliquest.ipmc.cnrs.fr/^56^. The length of the arrow in the alpha-helical representations is directly proportional to the hydrophobic moment. It was found in^57^ that if the hydrophobic moment is high, it denotes that the peptide has high penetration efficiency (it can easily kill/ inhibit its target). On a general note, it can be seen that the discovered AVPs have a high hydrophobic moment. It is very likely that these AVPs have good antiviral potential, and the same can be verified by chemically synthesizing them in laboratories. It can be observed that the length of the discovered AVPs are much smaller than their parent proteins. Hence, this tool efficiently identifies the core antiviral region of a given protein that is responsible for its antiviral activity. Moreover, we performed CD-HIT with a threshold of 0.9 on the AVPs found in each protein separately and tried to visualize their distribution with respect to the AVPs present in our training set. For this purpose, the isometric mapping technique has been used^58^. The 2D visualization of these data points is presented in Fig. 5, where it can be observed that the predicted AVPs and the AVPs present in the training set have similar distributions. Hence, the discovered AVPs are purported to show good antiviral activity, which can be confirmed by synthesis and experimental validation.

## Conclusion

In this work, the Deep-AVPiden model has been proposed for identifying AVPs in several protein sequences to accelerate the task of antiviral drug discovery. It is a deep learning model based on TCNs that predicts whether a given peptide is antiviral or not. It takes peptides (alphabetical strings) as input, converts them into feature matrices, and outputs a probability score for them, which is used to interpret their antiviral potential. In addition to this, we used depth-wise separable convolutions to build another computationally and space-efficient model called Deep-AVPiden (DS), which can be deployed on resource-constrained devices. The Deep-AVPiden and Deep-AVPiden (DS) models have an accuracy of 90% and 89%, respectively, which is much better than the existing classifiers’ performance.

Furthermore, a web app has been deployed at https://deep-avpiden.anvil.app/ where users can enter different proteins and find AVPs with good antiviral potential. After choosing fifteen antiviral proteins found in various mammals, plants, and fishes, our app discovered and presented some AVPs which are purported to have a good antiviral potential (subject to experimental validation and analysis). In the future, we would like to use other state-of-the-art sequence modeling techniques like transformers to build classification models. Also, designing a two-level multi-label classifier for AVPs can be considered. Such a classifier would predict whether a peptide is antiviral or not in the first stage and then classify it according to its target virus family in the second stage.

## Data Availability

The datasets analyzed during the current study will be made available upon reasonable request to the authors of this study.
